# Supplementary material for: Beyond SNP heritability: Polygenicity and discoverability of phenotypes estimated with a univariate Gaussian mixture model
Source: PLoS Genet. 2020 May 19;16(5):e1008612. doi: 10.1371/journal.pgen.1008612 (PMC7272101; doi:10.1371/journal.pgen.1008612)
Supplement: Attachment — Submitted filename: Reviewer_Response_HollandEtAl_PLOS_GENETICS_21Sep2019.docx [file pgen.1008612.s003.docx]

***Reviewer #1:***

*In this report, the authors proposed a general computitional method that can infer the genetic architecture underlying complex traits. An idea similar to LD score regression, using a mixture Gaussian distribution GWAS summary statistics have been combined with a reference population (1KG); a powerful method, as demonstrated by the authors, various interesting parameters and questions, such as the proportion of causal variants and their variance can be addressed. In real GWAS summary data analysis, a range of GWAS complex traits have been tested and consistent to previous reported results in other studies.*

*Technical comments*

*1 Similar ideas but using individual-level data had been proposed in Bayesian approaches [Nat Genet, Zeng, 2018]. In particular, their model assume the effect size is relavent to MAF. It is likely to be the case that the MAF and effect has certain correlation, negative or positive upon the scheme of the selection pattern. It is probably one of the reasons that some z-score distribution was not fitted as well as others in the manuscript.*

Response

========

We thank the Reviewer for pointing this out. We agree that the Zeng et al 2018 paper is of relevance. We evaluated the effect of MAF-dependent genetic architecture on accuracy of parameter estimates in simulations: see Table S1 in our Supplement (and also ref O.Frei et al, Supplementary Figure 10a). The results indicate small deviations in polygenicity and heritability estimates. In our future work, we will explicitly model MAF-dependence of causal effect sizes, using only GWAS summary data. We note that the work of Zeng et al, relying on raw genotype data, was restricted to ~400k SNPs; with summary statistics, we are working with ~10 million reference panel SNPs. Thus, it is not directly comparable. However, we have revised the text to include discussion of Zeng et al. See “Relation to other work” in the Supplement.

*2 The kernel of the method is the introduction of the mixture model that is composed of the causal and null components. In the multinomial pdf function Eq 29 in Supplementary notes, H_i is taken as the mean 2pq for the n_i snps in the bin. However, as k_i is a small number, say k_i =1, the 2pq of this specific causal locus will be very different from the mean 2pq of n_i snps in this bin. It is not really clear H_i in Eq 29 has been updated for k_i or always taken as H_i as approximation? In gut feeling, it may effect the estimation. However, the authors emphized that the real calculation was based on convolution,*

Response

========

The reviewer raises an important point. The concern is with the heterozygosities of the potential causal SNPs: do we properly take them into account? Yes, we do. But not only that, we also properly take into account their LD with SNPs having GWAS summary statistics.

(1) We bin typed SNPs (SNPs with z-scores) into an HxL grid. Then, for any grid element, (2) we bin the tagged SNPs into fairly fine LD-r^2 bins: the range 0.05-1 is divided up into w=20 or so bins.

In the extreme, we can consider each typed SNP individually, and enquire about how its z-score arises from causal beta values distributed among the reference SNPs it is in LD with (the SNPs tagged by the typed SNP).

The two levels of binning ((1) and (2) above) can be finessed until we get converged results for our three parameters -- and of course it not necessary to go all the way to the extreme scenario. As mentioned in the text, we ensured the binning in (1) and (2) was fine enough to give converged results.

From (1) above, we have, in a given grid element, selected typed SNPs in a narrow range of H and L. Conceptually, think of these SNPs as being just a single typed SNP. Now, the first crucial point is that we look at the reference SNPs our typed SNP is in LD with: we group those into very narrow LD bins (w=20 bins). Our model is that the typed SNP's z-score arises from noise and possibly some causal effects among the SNPs it tags through LD: a causal SNP might be in any one of those latter bins, there might be more than one causal SNP in any one of those bins, and many of the bins might have causal SNPs, and all of these distinct possible scenarios need to be accounted for. k_i is our random integer variable for the number of causal SNPs in bin i. The second crucial point is that within bin i, regardless of i, the heterozygosities of the binned reference SNPs will be in a very narrow range -- effectively, heterozygosity binning of the SNPs tagged by a given typed SNP comes along free with the LD r^2 binning of those tagged SNPs (again, we're conceptually just dealing here with a single typed SNP whose z-score we are trying to predict). The reviewer is concerned with the situation when k_i is small, particularly k_i=1 and that the heterozygosity of the potential causal SNP in bin i is not properly accounted for in our model. But because all SNPs in bin i have very similar heterozygosities, it is necessarily properly accounted for regardless of whether k_i is small or large: it is given by "H_i" in the variance expansion in the Gaussian component of our multinomial expansion of the z-score pdf, which carries over entirely to the Fourier version of the z-score pdf (the question could equally be raised there too). So in terms of accuracy of our model, this is not an issue. We should note that in the "M2" model of Zhang et al [Nat Gen Aug 2018], each of the potential causal SNPs in LD with a typed SNP is approximated as having LD with the typed SNP given by the mean for all of the tagged SNPs (the LD score of the typed SNP divided by the number of the SNPs in LD), and their heterozygosities are implicitly treated an average way, arising through the measured standard error of the beta_hat effect size. This has now been better explained in the manuscript.

*3 Eq 51. H_bar is taken as 0.2165, similar critics as in the last comment.*

Response

========

H_bar is calculated from the reference panel. We are estimating heritability in a probabilistic sense from a fraction pi1 of all SNPs using summary statistics, where the heritability per SNP is proportional to the SNP's heterozygosity. The model is that the causal SNPs are distributed randomly. Therefore, the overall estimated heritability is necessarily constrained to depend on the mean heterozygosity of all SNPs. It is true that insofar as this inaccurately reflects the mean heterozygosity of the actual causal SNPs, the end result will be in error, as we have pointed out, but it remains a best and reasonable approximation from the model at hand, about which we are explicit. The subsection “Narrow-sense Chip Heritability” has been moved from the Supplement to the main paper to help clarify this point.

*4 The LD score is estimated from the 1KG, which is presumed to be “health controls”. For case-control traits, the LD in the case-control sample will be different from “health controls”, in particular the effect size is large or the trait in under selection. Any consequence due to this leap?*

Response

========

We agree that this issue should be addressed. Effect sizes of individual variants on complex phenotypes are tiny, therefore allelic composition of each variant in case and control groups is very similar resulting in similar LD structure. There will likely be a very slight change in the LD structure for different phenotype-defined subgroups within a population. However, this will be no more than some third-order effect. Imprecision in the phenotype-specificity of the reference panel is currently not a limiting factor in GWAS analysis. We have now clarified this in the paper: last paragraph in the “Data Preparation” subsection in the Supplement.

*5 The estimation procedure should be better demonstrated in the main text, which is the kernel of the paper. As well as its step-by-step algorithm, and demo code should be provided for others to follow up.*

Response

========

We agree with the Reviewer and have moved much of the methodology presented in the Supplementary Material into the main paper, including the subsection “Parameter Estimation”. All code is publicly available on github:

<https://github.com/dominicholland/GWAS-Causal-Effects-Model>

-- reference 27, before the "Model PDF: Multinomial Expansion" subsection. (A user-friendly version of causal mixture model is maintained as a separate software project on

<https://github.com/precimed/mixer> )

*Minors*

*1 The main text and the supplementary notes are very similar to each*

*other.*

*2 References are inconsistent between the main and the supplementary*

*notes.*

*3 Many typos. The ms has not been lined well for the reviewer to tell*

*where those typos. In the supplementary materials it is also referred*

*to “Supplementary Materials”, page 16 second column.*

*4 Probably the author is from physics field, I guess. The authors used*

*some jargons such as Dirac delta function. However, according the sup*

*Notes, Eq 6-8, the Dirac delta function seems a standard normal*

*distribution instead*.

Response
========

We thank the Reviewer for these comments, and have revised the manuscript accordingly.

***Reviewer #2:***

*In this paper the authors present a method for estimating the genetic architecture of a complex trait from the set of GWAS summary statistics and an estimate of the LD matrix. From a methodological perspective, the major contribution of the paper seems to be the methods, presented in the supplement, for fitting the model using summary statistics and the alone. In the model, the marginal effect sizes for each individiaul SNP are assumed to be generated due to tagging effects of multiple linked SNPs, where the causal effects of the linked SNPs follow a mixture distribution that is a combination of a point mass at zero, and a normal distribution with some variance. Such mixture distributions are fairly common in the field, but one contribution here seems to be a conceptual stand on principle that a significant number of variants should have no effect on a given trait at all, and the methods here fit such a model (whereas most mixture distribution approaches assume that all sites makes at least some small contribution).*

Response

========

We agree that such mixture distributions have indeed become fairly common since we first -- in May 2017 -- introduced a causal mixture model (for causal beta values) and solved for the probabilistic distribution of summary statistics (z-scores):

[https://www.biorxiv.org/content/biorxiv/early/2017/05/24/133132.full.pdf](https://www.biorxiv.org/content/biorxiv/early/2017/05/24/133132.full.pdf)

That paper describes the correct multinomial expansion for the likelihood function, and was the first to employ an extensive reference panel. The current method was also presented on BiorXiv in June 2018, and this version is now cited by some of the published papers that use Gaussian mixture distributions for causal effects. We agree that at this stage the major contribution is in the methods: the underlying model is easy to state, but implementing it correctly and efficiently to produce a faithful pdf for the GWAS  z-scores, and solve correctly for the model parameters, is quite  involved and prone to missteps. We have clarified how our work contributes to the field in the revised manuscript, and, reflecting the importance of the methods, moved much of that material from the Supplement into the paper.

*In terms of the presentation, I think it would be helpful if the authors could present a bit more of the details of the model could be presented. E.g. I see no reason why equations 5 and 16 from the supplement (along with perhaps whatever others the authors feel necessary to provide appropriate context; see the following) couldn't appear in the main text. More broadly, I think the paper could do more to help place the present work in the context of existing methods of a similar flavor (of which there are many). What specifically distinguishes the approaches laid out here from those which can be found elsewhere in the literature. The reader shouldn't have to take a detour to read 10 other paper in order to figure it out.*

Response

========

We agree that the presentation would improve as the reviewer suggests. In the revised manuscript, much of the pertinent material in the Supplement has been moved to the main text, which is now fairly self-contained (i.e., not requiring reference to the Supplement in order to be comprehensible). We have added a subsection at the start of the Supplement describing the relation to some well-cited recent publications.

Our work provides unique contributes to the field, and we have now clarified this in the revised manuscript. There is similarity between our work and the reports of others. Zhu and Stephens [89] implemented a similar causal mixture distribution, but did not take the possibility of inflation into account. They use a reference panel to estimate LD between SNPs, but restrict it to the specific GWAS SNPs, i.e., the ones for which summary statistics are available. Thus, for height, for example, their causal beta values perforce are restricted to the typed GWAS SNPs (~1 million), whereas we use a much larger reference panel (~11 million) and allow the z-scores in the GWAS of interest to arise from any of the reference panel SNPs. They take care to allow for correlated "noise" in the estimated GWAS effect sizes. Their maximization scheme for the likelihood function involves Markov chain Monte Carlo, and is therefore computationally intensive.

The "M2" model of Zhang et al [Nat Gen Aug 2018] also uses a causal mixture Gaussian similar to ours. They employ a reference panel of ~1 million SNPs, and allow the causal SNPs to be any of those not necessarily (typed) GWAS SNPs; thus, for GWASs with summary statistics for SNPs *not* in the reference panel, they must restrict the z-scores analyzed to be from SNPs in the reference panel, but the causal SNPs can be any of the reference SNPs. (In our case, the reference panel is sufficiently large that no such restriction is required.) Their analysis is based on an inaccurate expression for the effective beta at a focal SNP arising through LD from underlying causal beta values at neighboring SNPs (they did not take into account the heterozygosity of those neighboring SNPs – “Hj” in the "equations 16" referred to above), and their likelihood function ignores the detailed LD and heterozygosity structure of the focal SNP, which we describe how to implement in a controlled and accurate way using either Fourier methods or a multinomial expansion (where we take w_max=20 in our "Model PDF" section, they take w_max=1) – see our response to Reviewer#1’s second comment; our Fourier approach is essentially exact, and fast. As a result, their "M2" model solutions do not accurately reflect the capability of the underlying model, i.e., their parameter estimates are inaccurate; their "M3" model, which adds an extra Gaussian, is implemented in the same fashion. The inaccuracy in the M2 model can be seen in the QQ plots (even though they are fairly restricted, with the max value for -log10(p) set to 10) for, e.g., height, LDL cholesterol, total cholesterol, years of schooling, Crohn's disease, coronary artery disease, and ulcerative colitis, for which we obtain much better fits. In simulations, we set w_max=1 for a few cases and estimated the model parameters. Example(A): for pi1=1e-4 and h^2=0.1 (with sigb^2=4.3e-4), we obtained pi1_hat=4.5e-5, h_hat^2=0.036 (with sigb_hat^2=3.4e-4) with w_max=1; running our precise model, we obtain pi1_hat=1.03e-4, h_hat^2=0.10 (with sigb_hat^2=4.20e-4) -- see Supplementary Figure S3. Example (B): for pi1=1e-3 and h^2=0.4 (with sigb^2=1.68e-4), we obtained pi1_hat=4.7e-4, h_hat^2=0.15 (with sigb_hat^2=1.3e-4) with w_max=1; running our precise model, we obtain pi1_hat=0.93e-3, h_hat^2=0.39 (with sigb_hat^2=1.75e-4) -- see also Supplementary Figure S3.

Exploring potential MAF-dependence on causal effect sizes, using only GWAS summary data, will be a focus of future work. However, for this basic model, we have explored it regarding model-misspecification: see our response to Reviewer#1’s first comment, and Table S1 in the Supplement. At this stage, our concern is to present the basic model, correctly implemented and analyzed. We note that the work of Zeng et al [Nat gen 2018], using a related causal distribution for true effects beta but with MAF-dependence, and requiring raw genotype data, was restricted to ~400k SNPs; with summary statistics, we are working with ~10 million reference panel SNPs.

*I must confess I am a bit concerned about identifiability issues here. E.g. the authors show in the supplement that if you simulate data under diffiferent assumptions, you can somewhat seriously misestimate the number of causal SNPs. In that specific case (where effects come from a particular mixture of Gaussians), the show that their model does not fit the QQ plot well, so we might take the fact that the the model does fit the QQ plots for real traits as a reason to believe in the estimates, but this is weak evidence at best. In the one case where the authors have real data for the same trait at multiple different samples sizes (height), the estimated number of causal SNPs is correlated with GWAS sample size. It is of course hard to know exactly what to make of this, given the known structure confounding issues in the GIANT consortium data, but it is not super reassuring. If the true effect size distribution exhibits the sort of long flat tails the emerge from scale mixtures of Gaussians (i.e. the sort that is explored in the supplement), I would not be surprised to see sample size dependence of parameter estimates, particularly the polygenicity, if the effect sizes truly follow this distribution.*

Response

========

We agree with the reviewers that this is an important issue. The 2010 and 2014 height GWASs have sample sizes 133k and 251k. Thus, both are quite large, with the second almost twice the size of the first; additionally, the 2010 GWAS appears to be deflated while the 2014 GWAS appears to be considerably inflated, and *still* we get remarkably consistent results. We are of the opinion that the consistency of the model results for these substantially different GWASs -- estimated number of causal SNPs 4,800 and 6,200, respectively, out of a total of ~11 million SNPs, and variance of causal effects sigb^2 equal to 1.44e4 and 1.23e4, respectively (even though across phenotypes we find sigb^2 ranging by more than two orders of magnitude) -- points to (1) the relevance of the model, and (2) that it is correctly implemented and solved. The 2018 height GWAS has sample size ~707k, and appears to have far more issues with population structure. It should be noted that we not only present the summary QQ plots, but also give a 4x4 grid of Total LD x Heterozygosity breakdown (from an overall 10x10 grid) for each phenotype in the Supplement, showing reasonable consistency – something other researchers have not done.

*In general, I don't like to suggest too much extra work in review, but it strikes me that it would be reassuring to see something like the UK biobank height data subsetted to different sample sizes, as a demonstration that you get consistent estimates of the number of causal sites. I.e. if you run 10 GWAS on resampled subsets of 50k UK Biobank participants, and then apply your method, do you get roughly the same average estimates for the parameters as you do if you run 10 GWAS on different subsets of 200k individuals?*

Response

========

We include new results for UKB_GB_2017 with sample size 336k, restricted to the GIANT SNPs. We find n_causal=7,800 and sigb^2=1.3e4. See Supplementary Figure S10. The scenario the reviewer outlines would be reasonable for uncovering pure sample-size dependency in our pi1 estimates. However, for the reasons discussed above we do not believe this is an important issue. For extremely large sample sizes, complicated population structure will increasingly play a role – and analyzing that is not in the scope of this paper.

*I also wanted to respond briefly to note that, while it is a very minor point in the context of this paper, I think the authors have a mistaken view of what the omnigenic model is. The omnigenic model, as articulated by Boyle et al, and more recently by Liu et al, is not a claim about the total number of casual SNPs that exist for complex phenotypes. Rather, it is a conceptual model meant to explain multiple features that are commonly observed in GWAS data. One of these features is that the total number of causal SNPs is high, but I think those authors would argue that ~10k is still "high", as far as they are concerned. But another important feature is the fact that much heritability seems to be attributable to variants within genes that are not directly related to the the trait in question, but simply expressed in the relevant cell type. In fact, the "omni" in "omnigenic" is best interpretted in this context. It means roughly "all genes expressed in the relevant cell types affect the trait" not "all variants affect all traits", which is what I read the authors to be arguing against when they say in the main text that height is "very far from omnigenic".*

Response

========

We thank the reviewer for pointing this out. The text has been updated:

'The number of causal SNPs (out of a total of approximately 11 million) is approximately 6k; although this is about one twentieth the estimate reported in \cite{boyle2017expanded}, it remains large and allows for height to be interpreted as ``omnigenic''.'

*Is software to fit the model made available? I could not find any mention of it in the text.*

Response

========

Yes. All code is publicly available on github: <https://github.com/dominicholland/GWAS-Causal-Effects-Model> -- reference 27, before the "Model PDF: Multinomial Expansion" subsection. (A user-friendly version of causal mixture model is maintained as a separate software project on

<https://github.com/precimed/mixer> )

***Reviewer #3:***

*This manuscript describes a new method for estimating heritability and the number of causal SNPs for GWAS traits. The proposed method relies on a spike-slab model and perform a grid search based on MAF and LD pattern for inference. The authors applied the model to analyze 16 diverse phenotypes including eight binary disease traits. Overall, the manuscript is well written, and the analysis is well carried out. The propose algorithm makes sense and appears to work well. With that said, however, I do have a few main concerns:*

*1. The proposed spike-slab model is identical to that used in ref #14 and one of the proposed inference algorithms (the one based on the multinomial expansion) is also very similar to the one proposed in ref #14. Therefore, it would be important to provide a detailed description of the method in ref #14 and explain how the present method differs from that of ref #14. In addition, it would be important to perform comprehensive comparisons with the ref #14 method in all simulations and real data applications, to demonstrate any benefits of the proposed method.*

Response

========

We agree with the Reviwer that there is strong similarity between the underlying causal effects model which we originally presented in May 2017

<https://www.biorxiv.org/content/biorxiv/early/2017/05/24/133132.full.pdf>

and the "M2" model of Zhang et al [Nat Gen Aug 2018]. We have now explained this more in detail in the revised manuscript.

The "multinomial" inference algorithm we presented is mathematically correct with the degree of finesse, leading to converged results for our model parameters, being controllable, whereas the one presented in Zhang et al is approximate. See the response to Reviewer #2 above. The "multinomial" approach of Zhang et al [Nat Gen Aug 2018] reduces, for their "H"=1 (single causal Gaussian), to a binomial for two categories of SNPs: causal and non-causal. All causal SNPs in the “M2” of Zhang et al are treated the same regardless of their LD with the focal SNP (the one with a z-score they are interested in predicting). This is mathematically incorrect: their binomial produces only a rough approximation to the full multinomial that we described in May 2017.

We have now explained this more in detail in the revised manuscript – see in particular “Relation to other work” in the Supplement. In Supplementary Table 6 we provide a comparison of our results with those for "M2" on real phenotypes.

*2. The authors did not provide any software. Some of the proposed algorithms are complicated. Without providing an open-source and user-friendly software along with a software manual, it would be almost impossible for anyone to use the proposed method.*

Response

========

All code used in this paper, including for simulations, is publicly available on github:

<https://github.com/dominicholland/GWAS-Causal-Effects-Model>

-- reference 27, before the "Model PDF: Multinomial Expansion" subsection. (A user-friendly version of causal mixture model is maintained as a separate software project on

<https://github.com/precimed/mixer> )

*3. The authors did not provide the analysis code used in the paper. I am thus unable to reproduce any of their simulation and real data analysis results. It would be important to provide all analysis code along with all analyzed gwas summary statistics.*

Response

========

All code used in this paper, including for simulations, is publicly available on github:

<https://github.com/dominicholland/GWAS-Causal-Effects-Model>

(Additional tools for simulated data are available here

<https://github.com/precimed/simu> )

*4. It has been shown previously that sparse models, like the spike-slab model used in the present paper, are not suitable for heritability estimation under polygenic settings. Indeed, sparse models often under-estimate heritability, at least in studies with small samples (e.g. as illustrated in ref #71). I am thus surprised to see the h2 estimates are fine in the simulations. I was wondering if this is due to the unrealistic simulations employed in the present paper: all simulations were based on simulated SNPs but not based on SNPs from real data. Therefore, it would be important to perform realistic simulations either using real genotype data or following the simulation approach used in ref #14.*

Response

========

The work of Zhou, Carbonetto, and Stephens [PLOS Genetics, 2013], apparently calling into question the suitability of point-normal (or sparse) models for heritability estimation under polygenic settings is not definitive, and certainly Zhou and Stephens (2017), Zhang et al (2018) and Zeng et al (2018) clearly find considerable utility in sparse models. The work of Zhou and Stephens on Regression with Summary Statistics (RSS) [Ann Appl Stat. 2017] provides an enlightening update. A lot depends on the details of the implementation. In future work, we will examine more sophisticated models for the distribution of causal effects. However, it is important to get the basic model right.

Regarding simulations, we agree that this is an important issue. An important point of Hapgen -- which we use -- is to be able to do realistic simulations, i.e., using realistic heterozygosities and realistic LD structures for SNPs. (For validation of our Hapgen genotype panel see Frei et al, Supplementary Figure 20.) However, any realistic simulations will also depend on the underlying model for distributing causal effects. SNP heritability estimates for phenotypes are generally less than heritability estimates from twin and family studies; they should be seen as providing a realistic lower bound for narrow-sense phenotypic heritability. It is not surprising that our simulations gave reasonable estimates for the SNP heritabilities: rather, they show that our implementation of the model is correct. Indeed, any underlying model for the distribution of causal effects, if solved correctly, should provide reasonable SNP heritability estimates. Nevertheless, it could be that for some real phenotypes there is a large number of causal SNPs that are distributed with very week effects (very small sigb^2), and the finite contribution of these to heritability would be underestimated by the current model.

*5. Half of the analyzed traits in the present study are binary disease traits. It is well known that likelihood-based approaches are not suitable for h2 estimation for case control studies, as likelihood-based method would bias the estimation of h2 due to case control ascertainment (e.g. ref #75). I am thus concerned whether the proposed method can be used for analyzing binary traits. It would be important to provide mathematical details on how the proposed model can accommodate case control studies. For example, do you need to incorporate the spike-slab prior to the liability threshold model? Do you need to develop moment matching type of algorithms for inference? It would also be important to perform comprehensive simulations under ascertained case control settings, like those have been done in ref #75.*

Response

========

We thank the reviewer for pointing out this important issue. The likelihood-based approach analyzed in Golan et al ["Measuring missing heritability: Inferring the contribution of common variants", PNAS 2014] is restricted maximum likelihood (REML) in GCTA, which assumes a polygenicity of 1, i.e., every SNP is causal. The issue of downward bias of large-sample REML estimates of SNP heritability due to over-ascertainment of cases in case-control studies is considered further in "Contrasting genetic architectures of schizophrenia and other complex diseases using fast variance-components analysis", Loh et al, Nature Genetics 2015, in the context of BOLT-REML, which assumes a mixture distribution of small ('spike') and large ('slab') effects. We have not systematically studied downward bias in the context of point-normal causal mixture models. We note, however, that the observed-scale heritability for schizophrenia in Loh et al, with 22,177 cases and 27,629 controls, is h^2_o=0.415. On the liability scale, assuming 1% disease prevalence, this translates as h^2_l=0.23. Loh et al, in light of Golan et al [PNAS 2014], calculated the liability-scale heritability from "phenotype correlation-genetic correlation" (PCGC) regression, a moments-based approach requiring raw-genotype data which produces unbiased estimates for case-control studies of disease traits. For this, their PCGC-estimated liability-scale heritability for schizophrenia, again assuming 1% disease prevalence, is h^2_g=0.27. Thus, the downward bias in this case appears to be about four percentage points; that is, without correcting for downward bias, the liability-scale heritability would have been an underestimate of almost 15% (of the unbiased heritability estimate). The degree of underestimation decreases for smaller sample sizes. Our estimate for the liability-scale heritability of schizophrenia, from a larger sample than in Loh et al, is 0.21. This at least would be consistent with downward bias operating in point-normal causal distributions, in a manner similar to that in GCTA and BOLT-REML. This would then translate into either an underestimate of the number of causal SNPs, and/or an underestimate of the variance of the distribution of causal effects. We will explore this issue in future work, and for now simply note the issue in the main text. There should be no concern that the method can be used to analyze binary traits using GWAS summary statistics (vs. raw genotype data); at worst, relatively small corrections would be needed, which likely could simply be handled by calibration. -- See the last page of the Discussion section.
