# Supplementary material for: Beyond SNP heritability: Polygenicity and discoverability of phenotypes estimated with a univariate Gaussian mixture model
Source: PLoS Genet. 2020 May 19;16(5):e1008612. doi: 10.1371/journal.pgen.1008612 (PMC7272101; doi:10.1371/journal.pgen.1008612)
Supplement: Attachment — Submitted filename: hollandEtAl_coverLetter_PlosGen12Dec2019.doc [file pgen.1008612.s004.doc]

DOMINIC HOLLAND, PHD	Center for Multimodal Imaging and Genetics

and dept. of NEUROSCIENCES

uNIVERSITY OF CALIFORNIA, SAN DIEGO

TEL: (858) 822-1776	9452 Medical Center Drive

Fax: (858) 459-5648	la jolla, ca 92037

email: dominic.holland@gmail.com

December 13, 2019

Dear Drs Zhu and Williams and Reviewer #3:

We have substantially revised and resubmitted our manuscript, *Beyond SNP Heritability*, for your further consideration as a research article in PLOS Genetics.

In the editors’ email to me of November 18^th^, you addressed three items:

1) Please fully address the original comment #1 of reviewer #3 by performing additional simulations. Please compare your approach with the best model between M2 and M3. The M2/M3 approach will identify the best model between M2/M3 rather than performing the two models separately. Because your model is almost identical with the model in M2/M3, with different implementations, we believe this comparison is essential.

2) Please also address the reviewer 2's concern that your proposed method "tends to produce a higher estimated number of causal SNPs than the previous M2/M3 methods and tends to estimate the heritability lower than the previous M2/M3 methods." In the Table 1 and Table S4, please add standard errors of parameter estimations.

3) We do not require to address the original comment #5 by additional simulations, although such simulations are welcome. However, some discussion is indeed necessary.

Reviewer #3 had stated:

(A) In the previous comment #1, I suggested the authors to perform a comprehensive comparison with previous methods developed in ref #14 (M2 and M3 methods) in all simulations and real data applications, due to the apparent similarity between the proposed method and the previous M2/M3 methods. The authors only criticized the previous M2/M3 methods being incorrect and applied M2/M3 models to real data. In the real data applications, the authors showed that the proposed method tends to produce a higher estimated number of causal SNPs than the previous M2/M3 methods and tends to estimate the heritability lower than the previous M2/M3 methods. Without simulations, however, it is unclear whether such difference observed in the real data is due to the inaccurate estimates from the proposed method or the inaccurate estimates from the previous M2/M3 methods.

(B) In the previous comment #5, I suggested the authors to perform simulations based on the liability threshold model to examine any potential downward bias in heritability estimation for binary traits. The authors explained that the estimates from the proposed method did produce downward bias (in the case of schizophrenia, about 15% downward bias). Because the estimates from M2/M3 models for all binary traits are all higher than that from the proposed method, I was wondering if the previous M2/M3 methods were not susceptible to case control ascertainment and thus could produce unbiased heritability estimates. It would thus be important to perform simulations based on the liability threshold model, to better understand whether the biased heritability estimation is a unique drawback for the proposed method, or whether it also applies to M2/M3.

Regarding (A) above, we did in fact provide results of two representative simulations comparing our model with the M2 model (our implementation of their implementation), discussed at the end of the 2nd paragraph on the first page of the earlier Supplement, in the first section titled *Relation to Other Work*, which was referred to in the main text and in our response to the reviewers. Contrary to the reviewer's statement, we did not "only criticize the previous M2/M3 methods being incorrect", we described mathematically why that is so and provided two representative simulation results arguing the matter. Nevertheless, we have now performed a complete set of such simulations, and include a new table in the Supplement presenting the results (Table S6, p. S14), and discuss these in the now-extended opening section of the Supplement, pp. S2-S3. Additionally, we have added an analysis of the remaining real phenotypes overlapping with M2 that we did not include earlier: results of these are in a new table (Table S7 p. S14), and also discussed on pp. S2-S3.

Regarding comparisons with model M3 mentioned in (1) above: the important matter, as we see it, is comparing our model and results with M2, not M3. We describe issues with M2, arguing that the results in “ref #14” are problematic. Adding an extra Gaussian (model M3) to patch up what is missed in model M2 – missed not because of inherent deficiencies in the posited underlying distribution of causal effects (beta-coefficients), but rather in the implementation, using an inaccurate probability distribution for the summary statistics arising from the causal effects – does not confidently enlighten us on the role of the second causal Gaussian. A comparison with M2 is appropriate (we have the same mathematical expression for the distribution of betas). A further comparison with M3 might only make sense if all were well at the M2 level. Since we have argued that this is not the case, we have not therefore performed such comparisons – they would only confuse matters further while only appearing to add clarification.

Regarding (2) above, we discuss the potential reasons for differences in estimated polygenicity and heritability between our implementation and that of ref#14 in pp. S2-S3 of the Supplement. In Table S4 p. S12 we have added confidence intervals for Table 1, and we have added standard errors to the old Table S4, which is now Table S5 p. S13.

Regarding (3) and (B) above, reviewer #3 notes:

"Because the estimates from M2/M3 models for all binary traits are all higher than that from the proposed method, I was wondering if the previous M2/M3 methods were not susceptible to case control ascertainment and thus could produce unbiased heritability estimates."

This is incorrect, and additionally the direct comparison should be with M2, not M3. We provide Supplementary Table S5, directly comparing our results with those of ref#14 (as well as ref#15). For ulcerative colitis, we get h2=11%, M2 gets 10%; for AD we get 15%, M2 gets 7%. MDD and Crohn's disease yield consistent estimates of h2 between our model and M2: 7% vs 9% for MDD, and 15% vs 17% for CD. There might be some downward bias in our model with respect to case-control data, and we discuss this in detail in the specific instance of schizophrenia on p. 15. If inflation is ignored (as is commonly done, including in ref#14), i.e., if we do not scale h2 by sigma0^2 consistent with ref#14, we get liability-scale h2=24% vs the supposedly unbiased "PCGC" value of 27% (from ref#87; PCGC uses raw genotype data); M2 reports h2=29% in this instance. It is hard to argue from these numbers that we have a major issue of case-control downward bias to address in our manuscript, all the more so given that we point out mathematical inaccuracies in the implementation of the M2 *a posteriori* pdf for z-scores. In the new Table S7 we provide a comparison of our results with M2 where we do not rescale discoverability and heritability by the inflation parameter. In this table, we also provide estimates from our implementation of M2, and describe all this on pages S1-S2 of the Supplement.

If additional analyses are needed at this stage, please let us know.

Sincerely,

Dominic Holland, Ph.D
